# *In Vivo* Investigation of the Effectiveness of a Hyper-viscoelastic Model in Simulating Brain Retraction

**DOI:** 10.1038/srep28654

**Published:** 2016-07-08

**Authors:** Ping Li, Weiwei Wang, Chenxi Zhang, Yong An, Zhijian Song

**Affiliations:** 1 Digital Medical Research Center, School of Basic Medical Sciences, Fudan University, Shanghai, People’s Republic of China; 2 Shanghai Key Laboratory of Medical Imaging Computing and Computer Assisted Intervention, Fudan University, Shanghai, People’s Republic of China; 3 Shanghai University of Medicine &Health Sciences, Shanghai, People’s Republic of China; 4 Department of Medical Physics, Shanghai Proton and Heavy Ion Center, Shanghai, People’s Republic of China

## Abstract

Intraoperative brain retraction leads to a misalignment between the intraoperative positions of the brain structures and their previous positions, as determined from preoperative images. *In vitro* swine brain sample uniaxial tests showed that the mechanical response of brain tissue to compression and extension could be described by the hyper-viscoelasticity theory. The brain retraction caused by the mechanical process is a combination of brain tissue compression and extension. In this paper, we first constructed a hyper-viscoelastic framework based on the extended finite element method (XFEM) to simulate intraoperative brain retraction. To explore its effectiveness, we then applied this framework to an *in vivo* brain retraction simulation. The simulation strictly followed the clinical scenario, in which seven swine were subjected to brain retraction. Our experimental results showed that the hyper-viscoelastic XFEM framework is capable of simulating intraoperative brain retraction and improving the navigation accuracy of an image-guided neurosurgery system (IGNS).

To minimize their impact on both healthy tissue and brain function, neurosurgical procedures involving tumor resection below the cortex require the surgeon to establish a surgical path to the tumor, which also causes brain retraction. Deformation, followed by tissue retraction and resection, usually causes severe misalignment in image-guided neurosurgery systems (IGNS) and renders their navigation completely unreliable. In addition, when correcting for brain resection, a prerequisite is to correct for the brain retraction. Therefore, the primary step for improving the accuracy of IGNS is to correct for brain retraction. Brain retraction caused by mechanical processes is a combination of brain tissue shear, compression and extension. Correcting for it requires modeling not only complex mechanical behaviors but also the brain tissue topological discontinuity. A variety of strategies^1^,^2^,^3^,^4^,^5^ have been reported to estimate the displacement caused by brain retraction, but this quantification has been proven to be challenging. One possible strategy that has been widely accepted is to simulate brain retraction behavior using a biomechanical model that is driven by sparse intraoperative data.

The most commonly used models are the linear elastic model^4^,^5^ and the poroelastic model^1^,^2^,^3^. In general, the linear elastic model is suitable for simulating small brain deformations through a quasi-static process with a low stress-strain rate^6^,^7^. The poroelastic model was used by Kaczmarek *et al*. in 1997 to model hydrocephalus with a larger deformation^8^. However, this deformation process usually lasts several days and is far slower than the brain retraction process. Miga *et al*. and Platenik *et al*.^1^,^9^ extended this model using mixed boundary conditions (BCs) to predict the retraction. Sun *et al*.^3^ also employed a linear poroelastic model to predict brain retraction that used two cameras to acquire the displacements of the retractors. Kyriacou *et al*.^7^ summarized the modeling techniques and tested them using a one-dimensional experiment. He suggested that if there was no specific requirement for interstitial fluid movement, a viscoelastic solid model, as proposed by Miller *et al*.^10^,^11^, may effectively model brain retraction. Miller *et al*.^12^,^13^ developed a step-wise hyper-viscoelastic constitutive model to account for both the tension- and compression-induced brain tissue deformation behaviors. However, to date, this promising biomechanical model has not been used to simulate brain retraction.

Sparse intraoperative data are imposed as boundary conditions to the biomechanical model to infer a volumetric deformation field. Some groups have used intraoperative Magnetic Resonance (iMR) directly^4^,^5^,^6^,^14^,^15^, which could provide high-resolution 3D volumetric images of brain tissue displacement. Others^1^,^2^,^9^ have used intraoperative measured pressure or a combination of measured displacement and pressure. In recent years, more and more researchers have tended to use measured displacements of the exposed brain surface as BCs because they are simple and convenient to acquire^2^,^3^,^16^,^17^,^18^,^19^,^20^. The exposed surface displacements could be acquired with cameras mounted on the operating microscope^3^,^20^ with an laser ranged scanner (LRS)^16^,^17^,^18^,^19^, or with a conoprobe^21^,^22^.

In our previous work^23^, we presented a framework for the compensation of brain retraction. In that framework, we used an LRS to track displacements of the brain retractor surfaces, which were used as BCs, and the extended finite element method (XFEM) to produce an accurate representation of the tissue discontinuity. The results of a brain phantom showed that the framework was a promising approach to handle tissue discontinuity. In this paper, we implemented a hyper-viscoelastic XFEM model to simulate intraoperative brain retraction. We used nodal displacements of the inner sub-face of retractors inserted into the brain to constrain the hyper-viscoelastic XFEM equations. The results were quantitatively and qualitatively assessed using seven live swine.

## Results

We illustrated a workflow for simulating intraoperative brain retraction using the hyper-viscoelastic XFEM model-based framework. We have organized the presentation of our evaluation results into two subsections. In the first subsection, we quantified specific comparisons between the calculations from the model and the measured quantities for a single subject. In the second subsection, we reported the predominant experimental data as averages.

### Workflow of the hyper-viscoelastic XFEM model-based framework

**Figure 1:**
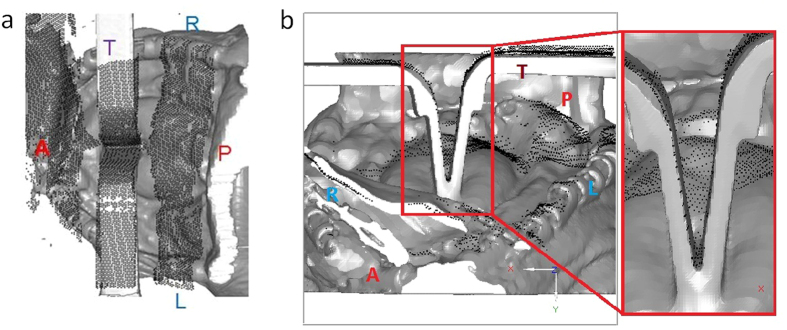
The LRS-captured point clouds (black dots) are perfectly aligned to the reconstructed surface of the postretraction brain (gray surface) when they are transformed into the same image space. (**a**) Top view. (**b**) Anterior view. The red box shows an amplified view of the retracted area.

Figure 1 shows the LRS-captured point clouds (black dots) and the reconstructed surface of the postretraction brain (gray surface). The point clouds were transformed to the postretraction image space. We can see that the LRS point clouds are perfectly aligned with the retractors’ surfaces, as well as with other parts of the surgical field, in the top (Fig. 1a) and anterior views (Fig. 1b).

**Figure 2:**
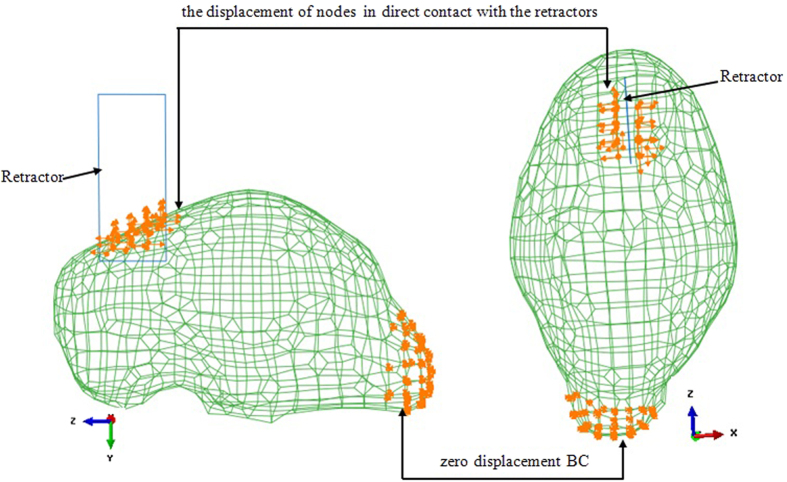
A typical BC distribution (subject 1). Two types of BCs were indicated. The zero-displacement BC is located in the brain stem. The other BC is the displacements of certain directly crack-related nodes.

Figure 2 is a pictorial representation of a typical BC distribution (subject 1). It illustrates various zones within the model that support different BCs. In the retraction region, the displacements of the surfaces in contact with the retractors are calculated by the brain retraction surface-tracking algorithm. In the brain stem region, traction-free conditions are prescribed, with no drainage.

**Figure 3:**
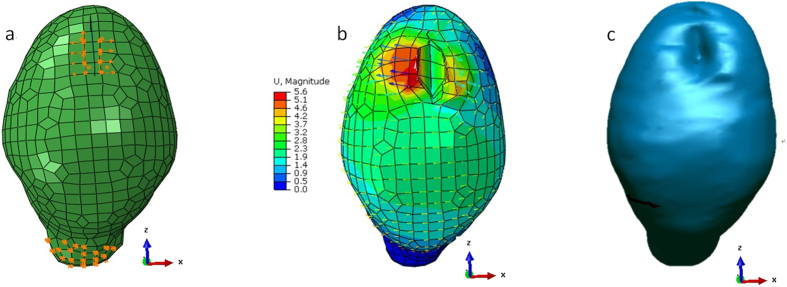
Hyper-viscoelastic extended finite element analysis results (subject 1). (**a**) The uniform preretraction hexahedral mesh (2115 nodes, 1663 elements) and the distribution of BCs. (**b**) Deformed mesh with colors corresponding to different magnitudes of displacement (red indicates the maximum and blue indicates the minimum). (**c**) 3D surface renderings of the predicted images by the ray-casting algorithm.

Two retractors were inserted into the brain of subject 1 and individually stretched to the right to a maximum distance of 5.4 mm and to the left to a maximum distance of 2.8 mm (The mean retraction was 7.1 mm). Figure 3 illustrates the results of hyper-viscoelastic extended finite element analysis (subject 1). Figure 3a is a pictorial representation of the BC distribution based on the volume mesh description. Figure 3b shows that the maximum displacement is found immediately around the crack. The arrows indicate the direction of brain retraction. Figure 3c shows 3D surface renderings of the predicted images.

### Evaluation of Simulation Accuracy for an Individual Subject

Table 1 shows the detailed comparisons of the landmark displacements using Euclidean distance measurement between the predicted images and the postretraction CT images. These comparisons indicate that the hyper-viscoelastic model predicted brain deformation displacement ranges between 1.1 and 2.5 mm; the actual measured deformation displacements in the postretraction CT images are between 0.9 and 2.9 mm. Similarly, the forecast errors of all landmarks varied between 0.1 and 0.9 mm (mean 0.4 mm), and the prediction accuracy between the predicted and actual results is between 63.8% and 94.8% (mean 80.5%).

**Figure 4:**
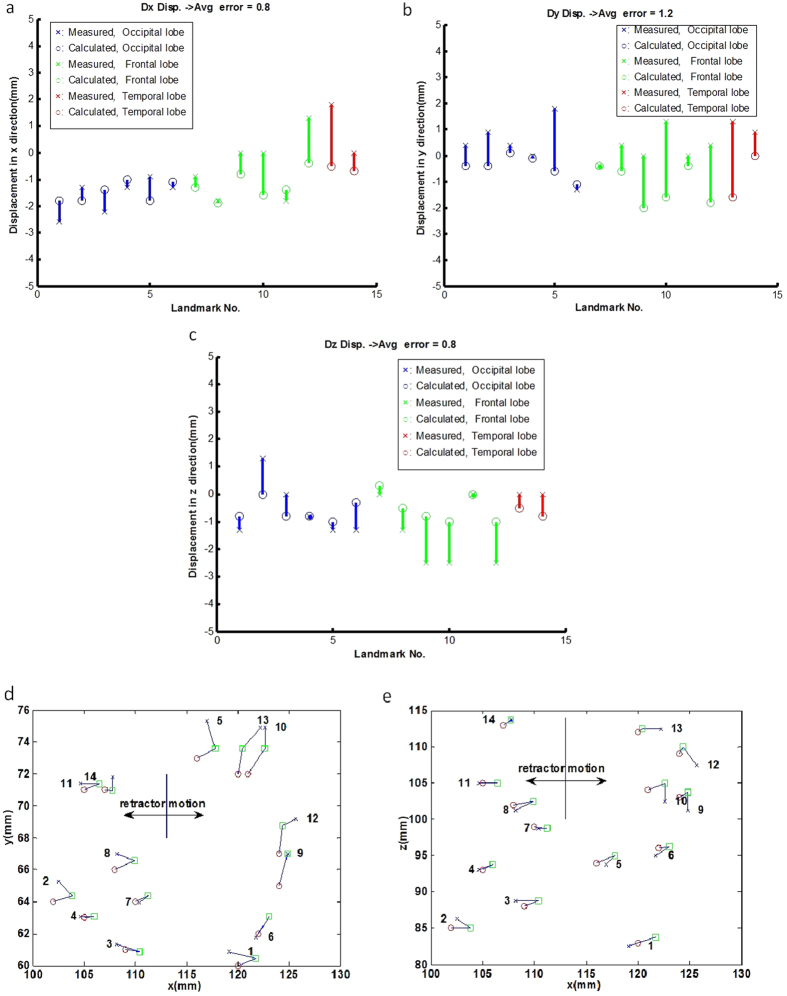
Comparisons of 14 landmark locations for subject 1. Comparisons of the X displacements (**a**), Y displacements (**b**) and Z displacements (**c**) between the actual measured displacements and the predicted displacements are shown for each landmark location. The coronal view (X-Y plane) (**d**) and axial view (X-Z plane) (**e**) of the comparisons between the preretraction and the measured and calculated postretraction landmark trajectories are shown for each landmark. □: preretraction, x: measured, o- calculated. The initial retractor position is represented by the solid line in each plane, and the direction of retraction is shown in both (**d**) and (**e**).

Figure 4a–c compares the actual measured displacements (from the postretraction CT images) with the predicted landmark displacements (from the hyper-viscoelastic model-based framework) in the X, Y and Z directions, respectively. Figure 4d,e show the comparisons of the preretraction and measured or computed postretraction landmark locations in different views for subject 1, providing a detailed pictorial analysis of our framework’s forecasted performance. Figure 4d shows a comparison of the coronal views (X-Y plane) between the preretraction and measured and calculated postretraction landmark locations. Figure 4e compares the preretraction and measured and calculated postretraction landmark displacements in the axial view (X-Z plane). Figure 4 indicates that the forecast error is not focused on specific frontal, parietal or occipital lobe regions. Detail statistical ANOVA tests were made to show whether the prediction accuracy and forecast error were influenced by landmark locations. The ANOVA results and their description can be found in the Appendix File.

**Figure 5:**
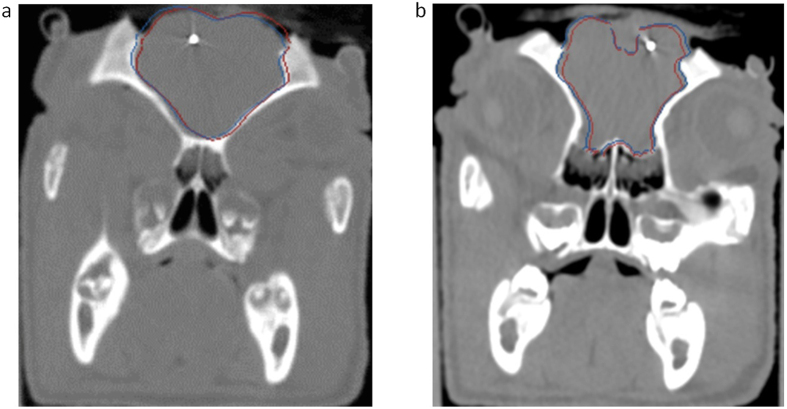
Comparisons of brain contours between the model-predicted image and the actual postretraction CT image (subject 1). (**a**) One slice of a preretraction image in the nonretraction area was overlaid with the corresponding brain contours of the model-predicted image (red) and the actual CT image (blue). (**b**) One slice in the retraction area. The brain contours of the model-predicted image and the brain edge of the actual CT image were identified separately and are shown as red lines and blue lines, respectively.

Figure 5 shows a comparison of the brain contours between the model-predicted and postretraction CT images. Figure 5a,b indicate that the images that were predicted by our framework (shown with a red line) agreed with the actual CT images (shown with a blue line). The two sets of contours agreed particularly well in two regions: the region in which the retractors were inserted and the region close to the brain stem.

### Evaluation of simulation accuracy for seven subjects

Model segmentation and mesh generation are time consuming. A computer (Intel Core i7-2600, 2.94 GB RAM) required 10 to 15 minutes to generate mesh models, which contained 2,018 to 4,622 nodes and 1,347 to 3,663 hexahedral elements, for each subject using a Windows 7 platform (Microsoft Inc., Redmond, WA). However, this did not delay the experiment because the procedure could be completed prior to the operation.

The comparisons of the forecast error and prediction accuracy of our framework for seven subjects are listed in Table 2. All subjects underwent retraction. The average prediction accuracy for all landmarks embedded in the seven subjects was 79.9 ± 15.2% (mean ± S.D.), and the average forecast error was 0.4 ± 0.4 mm (mean ± S.D.). The maximum average forecast error was 0.8 ± 0.7 mm (mean ± S.D.), which was observed in swine 5. The minimal average prediction accuracy was 71.5 ± 15.8% (mean ± S.D.), which was also observed in swine 5.

Table 3 shows comparisons of the calculation time, average prediction accuracy and forecast error between hyper-viscoelastic XFEM model and linear elastic XFEM model for seven swine. The comparisons were based on the same data. Solutions to linear elastic XFEM equations cannot be obtained for 5 of 7 subjects. For 2 of 7 successful subjects, the average prediction accuracy of the linear elastic XFEM model (83.7 ± 1.3% (mean ± S.D.)) is slightly higher than that of a hyper-viscoelastic XFEM model (81.7 ± 1.2% (mean ± S.D.)), while the average calculation time of the linear elastic XFEM model (35.5 ± 4.5 s (mean ± S.D.)) is longer than that of the hyper-viscoelastic XFEM model (25.5 ± 4.5 s (mean ± S.D.)).

Dice similarity coefficients for seven subjects are listed in Table 4. One can see from Table 4 that the average and maximum Dice similarity coefficients of all subjects were 88.9% and 94.5%, respectively. Table 5 shows the target registration error (TRE) for landmarks before and after model-update. The initial TRE is the distance between the landmarks in postretraction image and preoperative image. The TRE after model-update is the distance between the landmarks in postretraction image and model-updated image. The average initial TRE for all landmarks was 2.3 mm ± 1.2 mm (mean ± S.D.), while the average TREs after model-update was 1.1 mm ± 0.7 mm. The results suggest that the Hyper-viscoelastic XFEM model may decrease the misalignment between the intraoperative brain positions and preoperative image.

## Discussion

In this paper, a hyper-viscoelastic model-based framework was implemented to simulate brain retraction. The XFEM was used to model the crack resulting from brain retraction. An LRS-based retraction surface-tracking algorithm was introduced to acquire BCs for the hyper-viscoelastic model. The effectiveness of the framework was evaluated in seven swine. Using different evaluation methods, the hyper-viscoelastic XFEM model framework demonstrated sufficient capability to predict brain retraction, thereby improving the navigation accuracy of an IGNS.

In our framework, BCs were prescribed as the computed displacements of some specific nodes, and the calculated results of the brain retraction were also described as the displacements of the nodes. This type of problem has been described as displacement-zero traction or a pure displacement problem^24^,^25^. In this type of problem, not all parameters applied in the mechanical model could produce different results. However, the dimensionless ratio parameters for linear problems and a particular law for nonlinear problems do make a difference^24^. The linear elastic model, which has only one dimensionless parameter, the Poisson ratio, may be the best choice when modeling craniotomy-induced brain shifts due to its simplicity and widely accepted accuracy. However, the linear elastic model was not always sufficient for modeling brain retraction, based on our previous observations^23^ and current animal experiments. We have compared the linear elastic XFEM model and the hyper-viscoelastic XFEM model for prediction of brain retraction using the same data (Table 3). In all of subjects, solutions to linear elastic XFEM equations cannot be obtained for five subjects. For 2 of 7 successful subjects, the prediction accuracies of a linear elastic XFEM model were slightly higher than those of a hyper-viscoelastic XFEM model, while the average calculation time of the linear elastic XFEM model was longer than that of the hyper-viscoelastic XFEM model. One possible explanation is that brain retraction involving tissue compression and extension showed obviously nonlinear mechanical properties. This was not in accordance with the linear elastic theory but did agree with the nonlinear hyper-viscoelastic theory. Moreover, the energy function, together with its parameters that were systematically modified by Miller *et al*.^10^,^11^,^12^, provided a good representation of the mechanical behaviors of compression and extension.

In this paper, we used a portable LRS to track the displacement of the retractor surface points as BCs. The LRS was used to track the displacement of the cortical surface when compensating for craniotomy-induced brain deformation because it has been shown to be a cost-effective tool^16^,^17^,^18^,^19^,^26^. This method balanced time, BC quality and cost. IMR also can be used to track brain surface displacement. Although this method could obtain volumetric high-quality BCs, it was time consuming and expensive in the OR setting. When the measured pressures^2^,^9^ were used as BCs, some of the measurement settings in the surgical field were required to correct the pressure.

To evaluate our hyper-viscoelastic model, we implanted several stainless beads as landmarks for evaluation into the brains of live swine. The evaluation method was similar to that of previous reports^2^,^9^,^27^. However, unlike the work of Platenik *et al*.^2^,^9^ we also imbedded beads in the brain tissue, far away from the area of the V-shaped retracted brain tissue. The advantage of this modification was that we captured not only the quantitative results of the retracted brain tissue but also those of the brain far away from the retracted area. This could make our results more objective. However, as the retracted surface was far away from the retraction-free brain tissue, the displacements of the beads implanted in the retraction-free tissue were very small, making it difficult to obtain high-prediction accuracy in these positions. System errors, such as image resampling, image registration errors, and CT resolution, could have a significant impact on the evaluation of these landmarks. These results imply that the prediction accuracy would improve if only the landmarks in the retraction area were used for the evaluation. For example, for subject 5, the prediction accuracy would increase from 71.5% to 73.3%.

Two time point images were required for our framework: when the dura was removed and the brain shift had completed and when the brain retractors were positioned. Based on these two time point images, we used a single-step brain retraction model to simulate for the brain retraction, which was similar to the method used by Vigneron *et al*.^4^,^5^. In our framework, the process of obtaining BCs required several space transformations. If multiple errors in the transformations accumulated, this would possibly decrease the performance of the framework. Therefore, similar to Vigneron *et al*.’s claims^28^, we would ideally select the single-step brain retraction model to simulate brain retraction. Platenik *et al*.^9^ proposed an incremental model to compensate for brain retraction. In their framework, they used measured mixed BCs. Each single measurement was independent of the others. If even one measurement was mixed with noise, it is likely that the next step could decrease or even eliminate the noise.

The main limitation of our framework is that CT images were used as the gold standard, and we could not clearly distinguish the brain structures in these images, which degraded the performance of our brain segmentation. In future studies, we plan to use iMR to acquire the images, which will improve the brain segmentation. In addition, although we evaluated our hyper-viscoelastic framework in swine experiments, there are some differences between animal cases and human brain retraction cases. To make the experiment more convincing, we plan to use clinical cases to further test our framework. Furthermore, we will also try to identify a better evaluation method to enable evaluation in three or more dimensions.

The entire brain includes many other tissues, including the ventricles, falx, and brain-skull gap. In the future, we intend to investigate the framework of these tissues. Because the biomechanical features of these tissues differ, they should move in different ways upon retraction. By studying them, we can better understand how retraction in one region contributes to regional or even cross-hemispheric motion.

## Methods

The current study has been approved by the Animal Ethics Committees of Shanghai Medical College Fudan University. The methods were approved and supervised by the Institutional Animal Care and Use Committee at Fudan University. All procedures were carried out in accordance with the approved guidelines, including any relevant details.

### Brain retraction simulation framework

**Figure 6:**
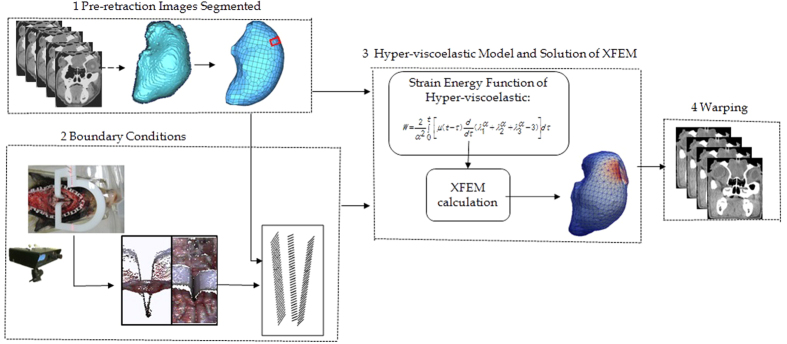
The hyper-viscoelastic model-based framework to simulate for brain retraction.

As shown in Fig. 6, the entire brain retraction simulation framework consisted of four steps. Each number represents one procedure. In step one, brain shift, which was caused by gravity or a craniotomy, was assumed to have been estimated accurately^29^,^30^. These updated images were regarded as the baseline preretraction images from which the brain was segmented. After segmentation, geometrical models of the brain tissue were acquired. Step two was designed to acquire BCs. One BC was the displacement of the crack-related nodes, which were determined by a brain retraction surface-tracking algorithm. The other BC was the zero-displacement area of the brain tissue. In step three, a uniform hexahedral mesh was first generated from a segmented brain using the octree algorithm^29^. A hyper-viscoelastic biomechanical model was then solved with XFEM equations to obtain the deformed mesh with available BCs. Finally, in step four, the deformed mesh was used to update the preretraction brain images with a modified back-interpolation algorithm, in which two-level sets are used to represent the crack^31^,^32^.

### Acquisition of BCs

Two types of BCs were required to achieve a unique solution to the XFEM equations. One was the zero-displacement BC. The displacements of the nodes at the brain stem were designated as zero. These nodes were manually selected by experienced surgeons. The other type of BC was the displacements of directly crack-related nodes in the operating field. This BC was calculated by a brain retraction surface-tracking algorithm^23^. These two types of BCs were imposed onto the hyper-viscoelastic model to achieve a unique solution and update the mesh, and the displacements of all nodes and crack-related information were obtained.

**Figure 7:**
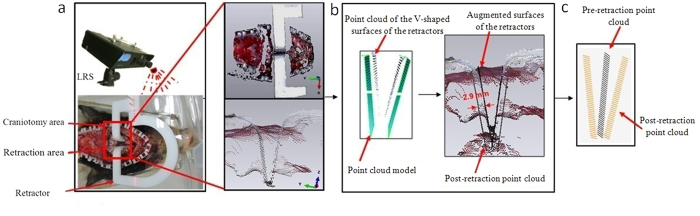
Surface tracking by LRS for subject 1. (**a**) The point cloud scanning procedure for the V-shaped surfaces of the retractors. (**b**) The point cloud model was registered to augmented retracted surface and extended a distance of 2.9 mm. (**c**) The point clouds of pre-retraction and post-retraction surface were transformed into the same image space and registered using CPD algorithm to obtain the displacements of retractors.

Figure 7 illustrates the steps for calculating the BCs using the surface-tracking algorithm. First, after retraction, the V-shaped surfaces of the retractors, was scanned using the LRS. Second, if the LRS failed to capture all point clouds of the inserted retractors, a previously prepared point cloud model of the retractors was registered to provide the missing part. The registered point cloud model was extended to a distance equal to the thickness of the retractor (2.9 mm) in the direction of the retractor surface norm (the green plane) to acquire postretraction surfaces. Third, the point clouds of the pre and postretraction surfaces were transformed into the same image space and registered using the CPD algorithm^33^ to obtain the displacements of the retractors. Therefore, the displacements of certain directly crack-related nodes were obtained as one type of BC. The time required to determine BCs depends on the manipulator’s skill. Generally, boundary conditions can be determined within 3 to 4 minutes.

### Hyper-viscoelastic model and XFEM equation solution

We used an Ogden hyper-elastic model^12^ to model brain retraction. To date, this promising biomechanical model has not been used to correct brain retraction. The energy function is as follows:


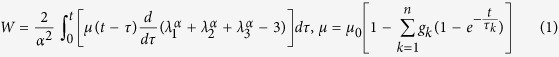


where *W* is a potential function, the *λ*_*i*_ variables are the principal stretches, *μ*_0_ is the instantaneous shear modulus in the undeformed state, *τ*_*k*_ is the characteristic time, and *g*_*k*_ is the relaxation coefficient. *α* is the material coefficient, which can assume any real value without restrictions. The material properties obtained from the eight swine brain experiments^12^ are listed in Table 6. In our experiments, a uniform hexahedral mesh with a mesh size of less than 5.0 mm was generated using the acquired geometric model of the brain tissue. The elements of the mesh were assigned hyper-viscoelastic properties.

The computation became complicated when the FEM was used to handle the discontinuity because the nodal shape functions (NSFs) were continuous functions^34^. The XFEM improved the FEM by adding extra degree of freedoms (DOFs) to the nodes that were related to discontinuity. This improvement made mesh adaptations^35^,^36^,^37^ or remeshing^31^,^38^ unnecessary. The XFEM displacement field is described as follows:


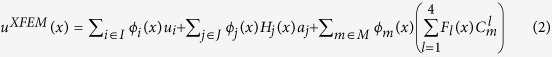


where *u*^*XFEM*^ is the XFEM displacement field. The first term on the right side represents the FEM displacement in which *I* is the set of FEM nodes, *ϕ_i_* is the FEM NSFs, *F*_*l*_ combines the radial and angular behavior of the asymptotic linear-elastic crack-tip displacement, and *u*_*i*_ is the FEM DOFs. To define the discontinuity for the XFEM, additional DOFs, namely *a*_*j*_ and 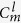, were added to sets *J* and *M*, which are subsets of set *I*. *H*_*j*_(*x*) is the Heaviside step function.

### *In vivo* experiment procedures

**Figure 8:**
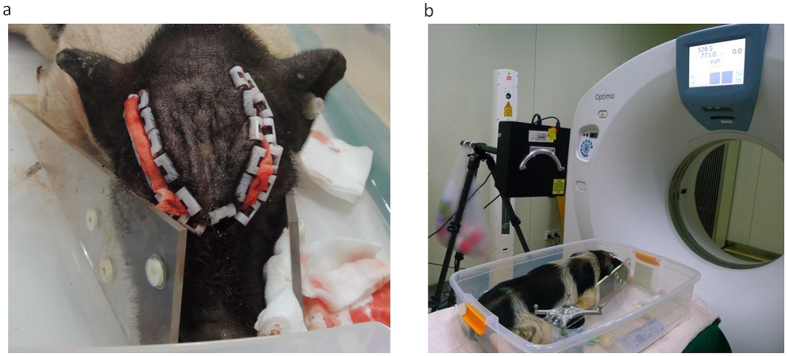
The swine experiment. (**a**) The swine was fixed in a Plexiglas box surrounded by 5 to 6 fiducial landmarks. (**b**) The swine experiment set-up with an LRS scanner in the CT room.

The swine experiments were similar to those performed by Paulsen’s and Miga’s groups^2^,^9^,^27^ and were conducted to quantify the fidelity of our framework. Seven 3-month-old subjects weighing approximately 15 kg were used. During the experiments, the subjects were fixed in a Plexiglas box to which 5 to 6 fiducial markers were affixed, as shown in Fig. 8.

(1) Preoperative CT Acquisition: Following anesthesia, a square region of the skull centered above the frontal-parietal lobes was removed, leaving the dura temporarily intact. Using a 14-gauge needle, 11 to 20 stainless-steel beads (1.5-mm in diameter) were implanted into the parenchyma, which were used as landmarks for evaluation. As determined by fluoroscopic imaging, two to six beads were separately embedded in the frontal, occipital and parietal lobes where retraction initially occurred. These embedded beads moved together with the brain tissue and worked as surrogates of the brain tissue movement. After this operation, CT images (Siemens SOMATOM Definition AS, Siemens Healthcare, Shanghai, China) were acquired with a spatial resolution of 512 × 512 × 421 mm^3^ and a voxel size of 1 × 1 × 1 mm^3^. These CT images, i.e., the preoperative CT images, were used to generate the mesh and construct the hyper-viscoelastic model.

(2) Intraoperative BC Acquisition: The exposed dura on the hemisphere designated for retraction was carefully removed. Two 14-mm wide and 2.9-mm thick Plexiglass retractor blades simulating NA20010 (JZ Surgical Instruments, Shanghai, China) were inserted into the hemispheric fissure to allow for bidirectional retractions laterally and away from the midline. The brain was deformed with a uniform force distributed along the retractors. An LRS and a brain surface-tracking algorithm were then applied to capture the displacements of some directly crack-related nodes. The postretraction brain images were predicted by our framework by constraining BCs in the retraction and zero-displacement areas.

### Method for evaluation of simulation accuracy

The postretraction CT images using the same CT scanner and scanning parameters served as a basis of comparison for the model-predicted images acquired from our framework. The evaluation methods were also divided into two types, which was similar to the method used by Li, *et al*.^23^. One type was the comparison of the displacements of the embedded landmarks, which was used to quantitatively evaluate the effectiveness of our framework. We compared the actual measured displacements (from the pre to postretraction CT images) and predicted landmark displacements (from the preretraction CT images to the model-predicted images). The forecast error is defined in formula (3). The simulation accuracy for every landmark is defined in equation (4).


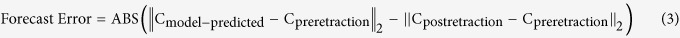



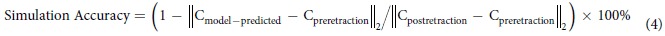


where 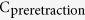, 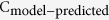 and 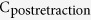 represent the landmarks’ coordinates in the preretraction, model-predicted, and postretraction CT images, respectively.

The other evaluation method compared the brain contours that had been segmented from the preoperative CT, aligned postretraction CT, and predicted images and was used to assess the morphological performance of our framework. In addition, in order to quantify the alignment degree between the contours obtained postretraction and those predicted by the hyper-viscoelastic XFEM model, dice similarity coefficient (DSC)^39^ was calculated for all subjects. The DSC measures the spatial overlap between two contours, A and B, and defined as DSC (%) = 2 (A ∩ B)/(A + B) where ∩ is the intersection. Moreover, we calculated target registration error (TRE) at the landmarks before and after model-update for quantitative assessment of the accuracy improvement of IGNS with the hyper-elastic XFEM model.

### Statement of approval

The current study has been approved by the Ethics Committees of Fudan University. All procedures were approved and supervised by the Institutional Animal Care and Use Committee at Fudan University.

## Additional Information

**How to cite this article**: Li, P. *et al*. *In Vivo* Investigation of the Effectiveness of a Hyper-viscoelastic Model in Simulating Brain Retraction. *Sci. Rep.* **6**, 28654; doi: 10.1038/srep28654 (2016).

## Supplementary Material

Supplementary Information

## Figures and Tables

**Table 1:** Comparisons of the actual measured displacements and predicted landmark displacements for subject1.

Landmark no.	Predicted displacement (mm)	Measured displacement (mm)	Forecast error (mm)	>Prediction accuracy (%)
1	2.0	2.9	0.9	66.9
2	1.8	2.0	0.2	91.7
3	1.6	2.2	0.7	70.4
4	1.2	1.5	0.3	82.5
5	2.2	2.3	0.2	92.9
6	1.5	2.2	0.7	68.8
7	1.3	1.0	0.4	63.8
8	2.1	2.2	0.1	94.8
9	2.3	2.5	0.2	92.2
10	2.5	2.8	0.3	88.2
11	1.5	1.8	0.3	84.9
12	2.1	2.9	0.8	72.3
13	1.7	2.2	0.5	78.6
14	1.1	0.9	0.2	79.0

**Table 2:** Comparison of the average forecast error and prediction accuracy for seven swine.

	Average Forecast error(mm) (mean ± S.D.)	Average Prediction accuracy(%) (mean ± S.D.)
Subject #1	0.4 ± 0.3	80.5 ± 10.3
Subject #2	0.3 ± 0.3	73.9 ± 19.9
Subject #3	0.3 ± 0.2	80.0 ± 13.8
Subject #4	0.4 ± 0.2	87.2 ± 27.2
Subject #5	0.8 ± 0.7	71.5 ± 15.8
Subject #6	0.4 ± 0.5	82.9 ± 18.4
Subject #7	0.4 ± 0.2	82.9 ± 10.7

**Table 3:** Comparisons of the calculation time, average prediction accuracy and forecast error between two models.

	Calculation time(s)		Average prediction accuracy (%)		Average forecast error (mm)	
	the Hyper-viscoelastic XFEM model	the Linear Elastic XFEM model	the Hyper-viscoelastic XFEM model	the Linear Elastic XFEM model	the Hyper-viscoelastic XFEM model	the Linear Elastic XFEM model
Subject #1	21	31	80.5	82.4	0.4	0.4
Subject #2	53	Fail	73.9	–	0.3	–
Subject #3	21	Fail	80.0	–	0.3	–
Subject #4	19	Fail	87.2	–	0.4	–
Subject #5	26	Fail	71.5	–	0.8	–
Subject #6	33	Fail	82.9	–	0.4	–
Subject #7	30	40	82.9	84.9	0.4	0.3

**Table 4:** Dice similarity coefficient for seven swine.

	Dice Coefficient (%)
Subject #1	90.2
Subject #2	85.3
Subject #3	92.6
Subject #4	94.5
Subject #5	82.8
Subject #6	88.5
Subject #7	88.5

**Table 5:** Comparisons of Average TREs for landmarks.

	Initial TRE (mm)	Model-updated TRE (mm)
Subject #1	2.1	1.8
Subject #2	1.4	0.8
Subject #3	1.7	0.8
Subject #4	3.1	0.8
Subject #5	2.0	1.4
Subject #6	3.1	0.9
Subject #7	2.7	1.0

**Table 6:** Material properties of the hyper-viscoelastic model used to model the mechanical response of the brain parenchyma.

Instantaneous response	*k* = 1	*k* = 2
*μ*_0_ = 842 *Pa*	*τ*_1_ = 0.5 *s*	*τ*_2_ = 50 *s*
*α* = −4.7	*g*_1_ = 0.450	*g*_2_ = 0.365
