# Supplementary material for: In Vivo Investigation of the Effectiveness of a Hyper-viscoelastic Model in Simulating Brain Retraction
Source: Sci Rep. 2016 Jul 8;6:28654. doi: 10.1038/srep28654 (PMC4937391; doi:10.1038/srep28654)
Supplement: Supplementary Information [file srep28654-s1.doc]

In Vivo Investigation of the Effectiveness of a Hyper-viscoelastic Model in Simulating Brain Retraction

APPENDIX PART

Ping Li^1, 2, 3^, Weiwei Wang^1,2,4^, Chenxi Zhang^1,2,*^, Yong An^1,2^, and Zhijian Song^1,2,*^

^1^Digital Medical Research Center, School of Basic Medical Sciences, Fudan University, Shanghai, People’s Republic of China.

^2^Shanghai Key Laboratory of Medical Imaging Computing and Computer Assisted Intervention, Fudan University, Shanghai, People’s Republic of China

^3^Shanghai University of Medicine &Health Sciences, Shanghai, People’s Republic of China

^4^Department of Medical Physics, Shanghai Proton and Heavy Ion Center, Shanghai, People’s Republic of China

The statistical Analysis of Variance (AVONA) tests were made to investigate whether the results are influenced by the beads locations. All the locations of beads were analyzed for seven objects in SPSS. The average predication accuracies of beads in the frontal lobe, temporal lobe and occipital lobeare 80.6±12.0%, 83.3 ± 14.1% and 74.0 ± 14.5% respectively. The average forecast errors of beads in the frontal lobe, temporal lobe and occipital lobe are 0.5±0.3 mm, 0.3±0.3 mm and 0.6±0.6 mm respectively. We can see from Table 1 and 2 that the significant levels are 0.061 (p=0.061) and 0.088 (p=0.088) respectively, which are larger than 0.05.

**Table 1** Relationship between prediction accuracy and beads' locations for seven swine

| **ANOVA Analysis** | **Sum of Squares** | **Difference** | **Mean Square** | **F** | **Significance** |
| --- | --- | --- | --- | --- | --- |
| **Among groups** | 1346.772 | 2 | 673.386 | 2.891 | 0.061 |
| **Within groups** | 18167.407 | 78 | 232.915 |  |  |
| **Total** | 19514.179 | 80 |  |  |  |

**Table 2** Relationship between the forecast error and beads' locations for seven swine

| **ANOVA Analysis** | **Sum of Squares** | **Difference** | **Mean Square** | **F** | **Significance** |
| --- | --- | --- | --- | --- | --- |
| **Among groups** | 0.815 | 2 | 0.408 | 2.511 | 0.088 |
| **Within groups** | 12.662 | 78 | 0.162 |  |  |
| **Total** | 13.477 | 80 |  |  |  |
